# The Nonlinear Dynamic Response of Intrapartum Fetal Heart Rate to Uterine Pressure

**DOI:** 10.22489/cinc.2022.268

**Published:** 2023-04-03

**Authors:** Johann Vargas-Calixto, Yvonne Wu, Michael Kuzniewicz, Marie-Coralie Cornet, Heather Forquer, Lawrence Gerstley, Emily Hamilton, Philip Warrick, Robert Kearney

**Affiliations:** 1 McGill University, Montreal, Canada; 2 University of California, San Francisco, USA; 3 Kaiser Permanente, Northern California, USA; 4 PeriGen Inc., Montreal, Canada

## Abstract

The research objective of our group is to improve the intrapartum detection of cardiotocography tracings associated with an increased risk of developing fetal acidosis and subsequent hypoxic-ischemic encephalopathy (HIE). The detection methods that we aim to develop must be sensitive to abnormal tracings without causing excessive unnecessary interventions.

Past studies showed that the dynamic response of fetal heart rate (FHR) to uterine pressure (UP) during the intrapartum could be modelled using linear systems. In this study, we examined the assumption of linearity by comparing the performance of linear dynamic and nonlinear dynamic models of the UP-FHR system. The linear systems were defined by second-order state-space models. The nonlinear systems were defined by Hammerstein models: a cascade of a static nonlinearity and a linear second-order state-space model.

Our results showed that nonlinear dynamic models were better than linear systems in 81.8% of UP-FHR segments.

## 1. Introduction

Neonatal hypoxic-ischemic encephalopathy (HIE) is a serious brain dysfunction often caused by intrapartum fetal hypoxia. Clinicians regularly monitor two signals during labor: fetal heart rate (FHR) and uterine pressure (UP) using cardiotocography (CTG). These signals are thought to carry information about the fetal state and how well the fetus is withstanding the stress of labor.

Warrick et al. demonstrated that the relationship between UP and FHR dynamics could be quantified using system identification [1]. Furthermore, they showed that the resulting system parameters could be used to identify fetal pathology [1]. To our knowledge, the identification of UP-FHR systems has been limited to linear dynamic models. However, physiological evidence suggests that the effect of uterine contractions on the blood flow in the uterine artery is nonlinear [2]. The uterine artery perfuses the placenta, bringing oxygen to be delivered to the fetus. Thus, there is a direct link between the blood flow in the uterine artery and fetal hypoxia. For that reason, we hypothesized that a nonlinear structure would model the UP-FHR system better than a linear structure.

## 2. Dataset and methods

### 2.1. Fetal database

We have access to the largest CTG database reported to date containing intrapartum signals for ~250,000 term births. Our database was collected at 15 Kaiser Permanente Northern California hospitals between 2011 and 2019. This study focused on two subsets of vaginal births: (1) 200 randomly selected normal cases with normal blood gas (pH > 7 and base deficit < 10 mmol/L); and (2) 170 HIE cases characterized by the presence of fetal acidosis (pH < 7 or base deficit > 10 mmol/L) at birth and clinical evidence of encephalopathy. Blood gas measurements were taken from the umbilical cord at birth or within the first two hours of life.

### 2.2. Signal pre-processing

The CTG signals were preprocessed using PeriCALM Patterns, a specialized software by PeriGen Inc. PeriCALM Patterns automatically repaired the CTG and identified important patterns such as the FHR baseline, FHR deceleration, FHR accelerations, UP contractions, and artifacts [3]. CTG was sampled at 4 Hz, high-pass filtered with a 4.5 mHz cut-off frequency, and decimated with antialiasing filtering to a 0.5 Hz sampling frequency.

After preprocessing, we removed the slow wave from the FHR signal that connected the baseline patterns, and one that connected the intervals between contractions within the UP signal. These slow waves were generated by linearly interpolating consecutive patterns and then smoothing the resulting signal with a moving average in a 360 second window. This centered the CTG signal amplitudes around zero for periods where there was no activity in the UP or FHR related to contractions.

CTG signals are noisy and often contain gaps related to disconnection or movement artifacts. We divided the signals into continuous 20-minute segments. To maximize the number of segments that could be used, we also included shorter segments at least 10 minutes long, and longer segments to a maximum of 30 minutes. Then, noisy samples, labelled as artifacts by PeriCALM Patterns, were removed from segments. Any resulting segments shorter than 10 minutes or with more than 20% noisy samples were discarded. The remaining segments were used for system identification.

### 2.3. System identification

For each segment, we estimated and compared the performance of two models: (1) a linear structure (LS) consisting of a 2^nd^ order linear state-space (SS) model and (2) a nonlinear Hammerstein structure (NLS) comprising a static nonlinearity, described by a fifth order Chebyshev polynomial, followed by a 2^nd^ order SS element. The LS models were identified using a subspace method, similar to [4]. The NLS models were identified using an iterative subspace algorithm for the identification of Hammerstein systems [5]. Identification was deemed to have failed for a segment if an algorithm did not converge or generated an unstable model.

### 2.4. Comparison of model structures

We used three criteria to assess the performance of the models:

1. the percent variance accounted for:
$$VAF\%=\left[1-\frac{\sigma^{2}\left(FHR_{\text{obs}}-FHR_{\text{pre}}\right)}{\sigma^{2}\left(FHR_{\text{obs}}\right)}\right]*100\%$$
  where *σ*^2^ is the variance, *FHR*_*obs*_ is the observed FHR, and *FHR*_*pre*_ is the predicted FHR,
2. the Akaike information criterion:
AIC=N∗log(SSE)+2∗M
  where *N* is the number of samples, *SSE* is the sum of squared errors of the prediction, and *M* is the number of free parameters; and
3. the minimum description length:
$$MDL=\left[1+M\frac{\text{log}(N)}{N}\right]*SSE.$$

The *VAF*% quantifies the accuracy of the predictions of the identified models. It is useful to compare models with the same structure. However, the VAF% does not account for the number of free parameters in a model. In this study, the LS has *M* = 10 parameters, while the NLS had *M* = 15. Models with more free parameters can overfit the output and model the noise, increasing the *VAF*%. The AIC and the MDL account for this by including the number of free parameters in their criteria.

To compare the LS and NLS models, we used the difference Δ*AIC* = *AIC*_*NLS*_ − *AIC*_*LS*_ and the ratio $MDL_{\text{ratio}}=\frac{MDL_{NLS}}{MDL_{LS}}$ A value of Δ*AIC* < 0 or *MDL*_*ratio*_ < 1, indicated that the NLS was better than the LS. Usually, *MDL*_*ratio*_ favors model simplicity while Δ*AIC* favors accuracy. Thus, if a structure is favored by both criteria, it is a more accurate model without being overly complex.

### 2.5. Delay estimation

UP signals are usually acquired using an abdominal CTG sensor that has an inherent delay in the detection of the pressure wave associated with contractions. This delay depends on many factors: sensor position, the thickness of the maternal abdomen, et cetera. In addition to the acquisition delay, there is also a physiological delay associated with the response time of the FHR to uterine contractions. To find the best delay we performed separate LS and NLS identification for UP delays ranging from −80 to 80 seconds in steps of 2 seconds. The system and delay with the highest *VAF*% was selected as the best model for each structure.

### 2.6. Surrogate analysis

The validity of the best LS and NLS models for a segment was evaluated using a surrogate test. Thus, for each segment, we generated 200 FHR surrogates using the amplitude adjusted Fourier transform (AAFT). The AAFT preserves a signals magnitude spectrum and amplitude distribution while randomizing its phase. This phase randomization destroys any causal UP-FHR relationship. The LS and NLS identification was repeated for each surrogate to generate a distribution of *VAF*% for unrelated signals (i.e., the system modelled the noise). If the *VAF*% of the original model was larger than the 95^th^ percentile of the surrogate *VAF*%, we considered the original to be valid with a significance of 95%.

## 3. Results

### 3.1. System identification results

**Figure 1:**
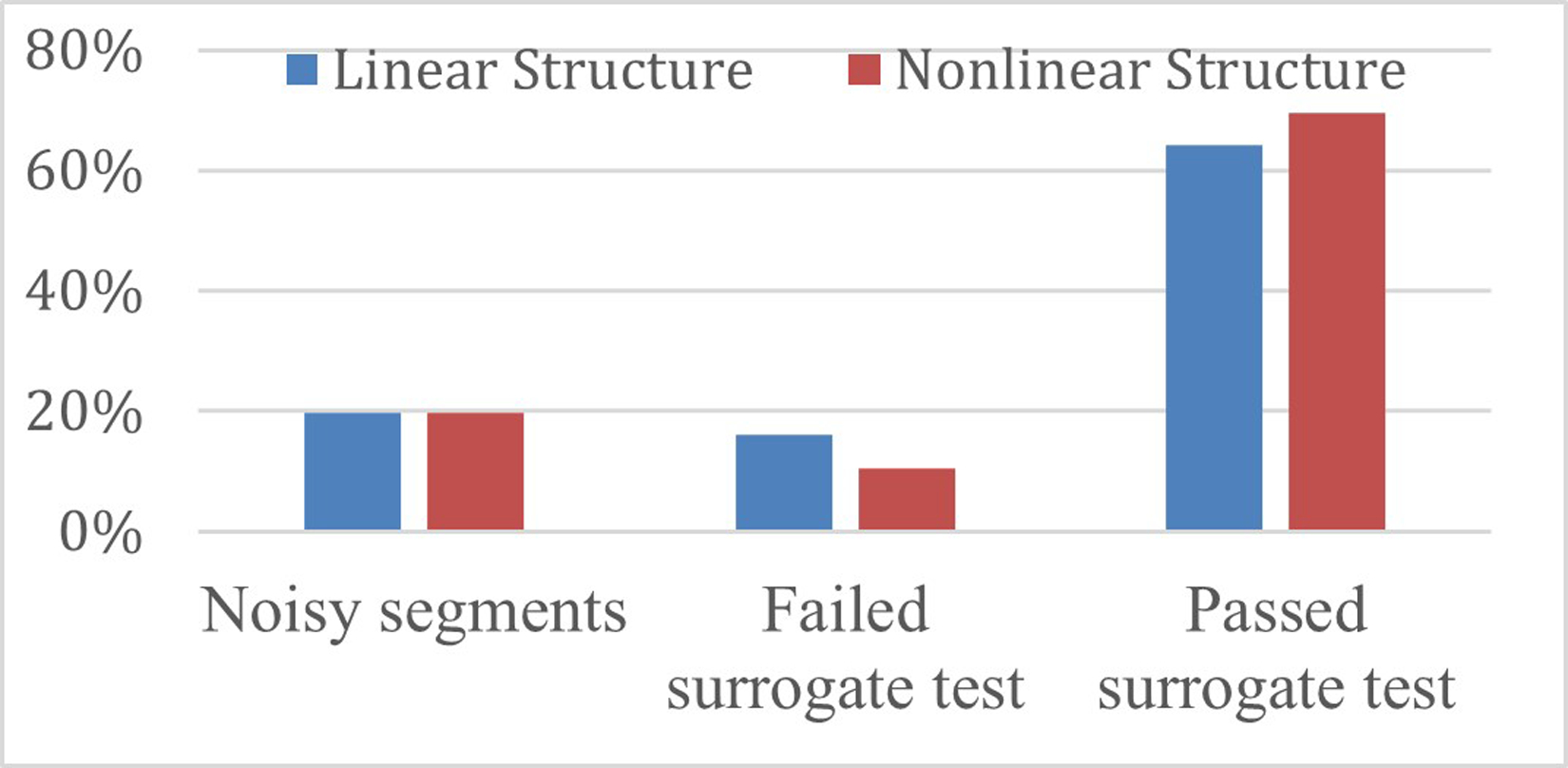
Distribution of segments according to the results of pre-processing, identification, and the surrogate test using linear (blue) and nonlinear structures (red).

Figure 1 shows the breakdown of model estimation results. There was a total of 8674 CTG segments, of these 1716 were discarded due to excessive noise. Identification failed in less than 0.1% of the remaining segments, due to convergence or stability issues. The number of segments where the identification was successful and yielded models that passed the surrogate test was larger for the NLS structure (6043) than for LS (5566).

### 3.2. Valid models

**Figure 2:**
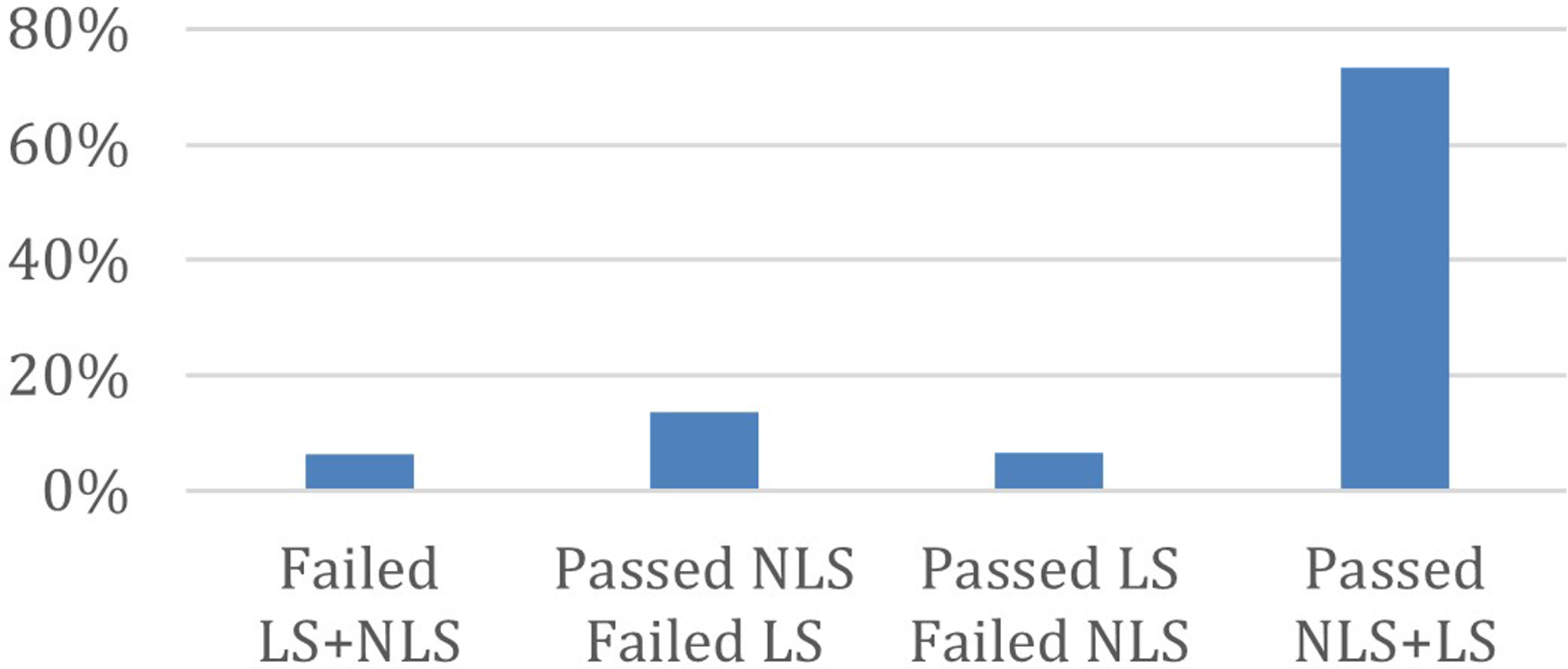
Number of segments for which the linear (LS) and nonlinear models (NLS) passed the surrogate test.

Figure 2 shows the proportion of segments for which the LS and NLS models passed the surrogate test. For most segments (5100), both model structures passed the surrogate test. However, the NLS model passed the test while the LS failed for 943 segments; and the LS model passed while the NLS failed in 466 segments. Finally, both LS and NLS models failed the surrogate test for only 449 the segments. The remaining analyses will focus on the 5100 segments for which both model structures passed the surrogate test.

**Figure 3:**
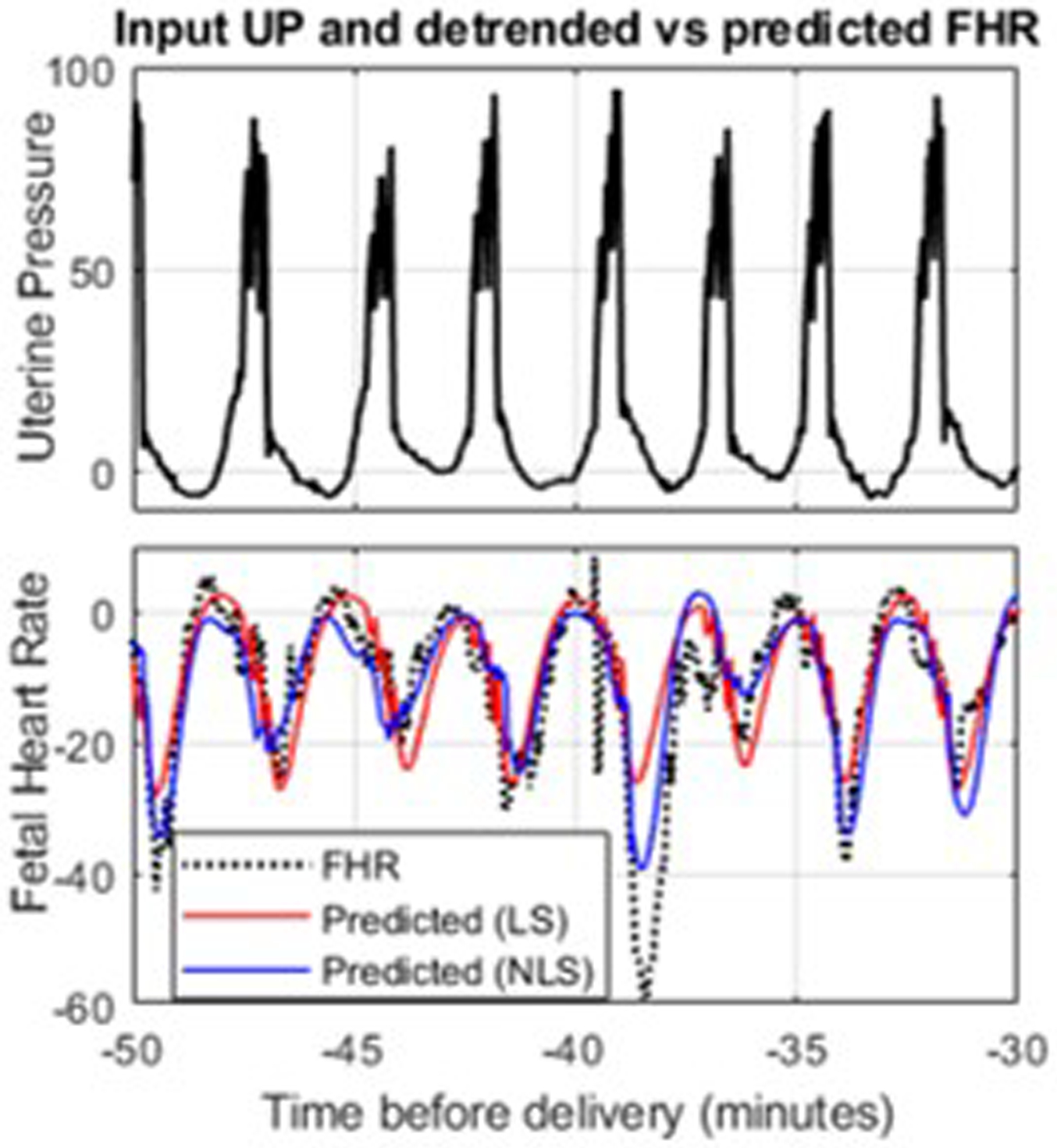
FHR and UP signals recorded for a typical HIE segment with the FHR predictions of the best linear (red; *VAF*% = 46.7%) and nonlinear models (blue; *VAF*% = 68.1%) predictions.

Figure 3 shows an example where the nonlinear prediction (blue) tracked the FHR better than the linear prediction (red). For this segment, the NLS *VAF*% was 68.1%, while the LS *VAF*% was 49.7%. Specifically, Figure 3 shows that the NLS predicted better the deeper decelerations at −49, −39, and −34 minutes before delivery.

**Figure 4:**
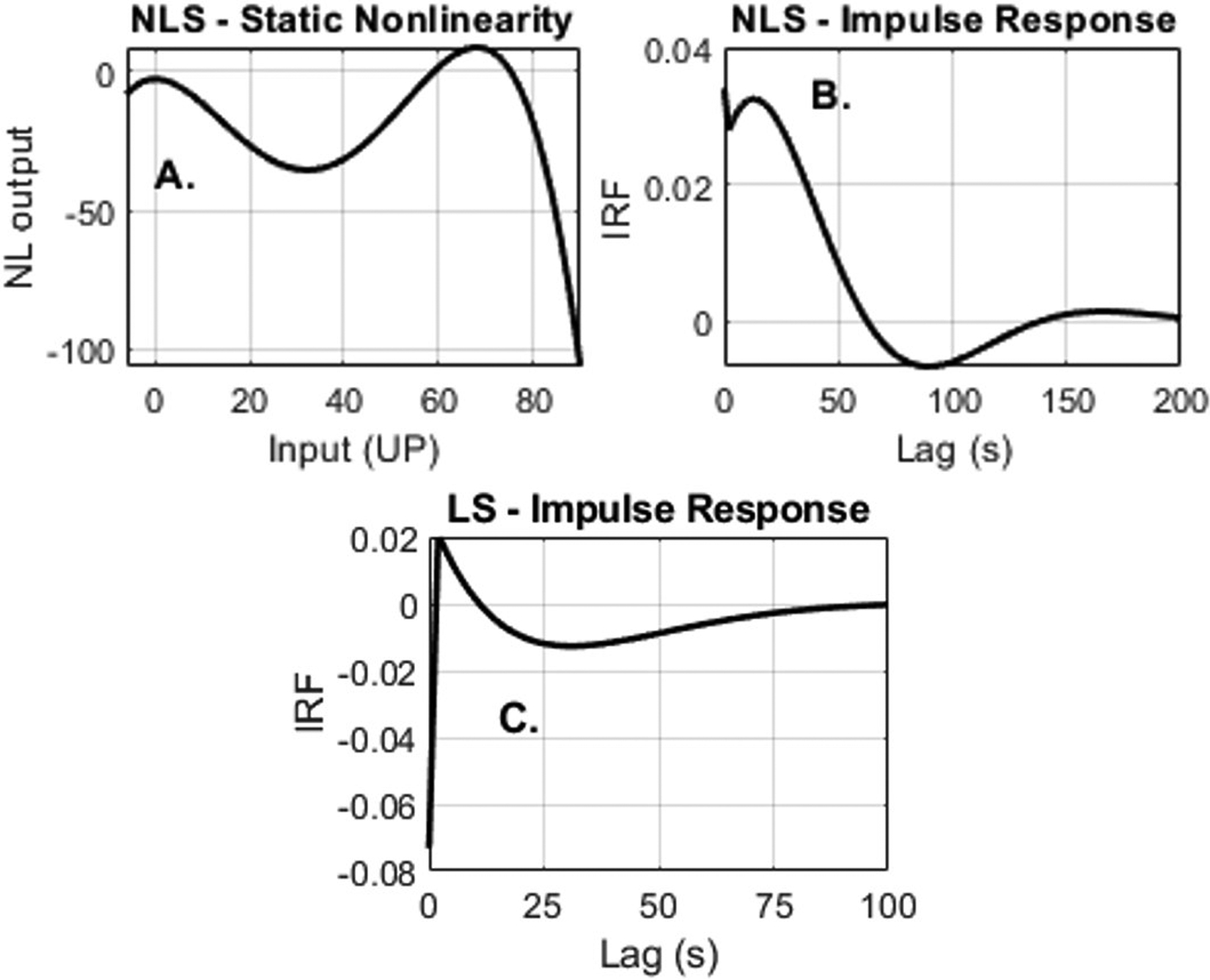
Nonlinear structure (NLS) comprising (A) a static nonlinearity and (B) linear dynamics; (C) linear structure identified for the segment in Figure 3.

Figure 4 shows the NLS (A, B), and LS (C) models identified for the segment in Figure 3. Figure 4 (B) and (C) show the impulse response of the SSM elements. The linear element of the Hammerstein model was normalized to have a steady state gain of 1. The static nonlinearity in Figure 4 (A) has a shape that generates small responses for low amplitude perturbations. However, perturbations that are larger than 60 bpm are amplified. This effect is evident in Figure 3 where the deeper decelerations are predicted better by the NLS than the LS.

### 3.3. Model selection

**Figure 5:**
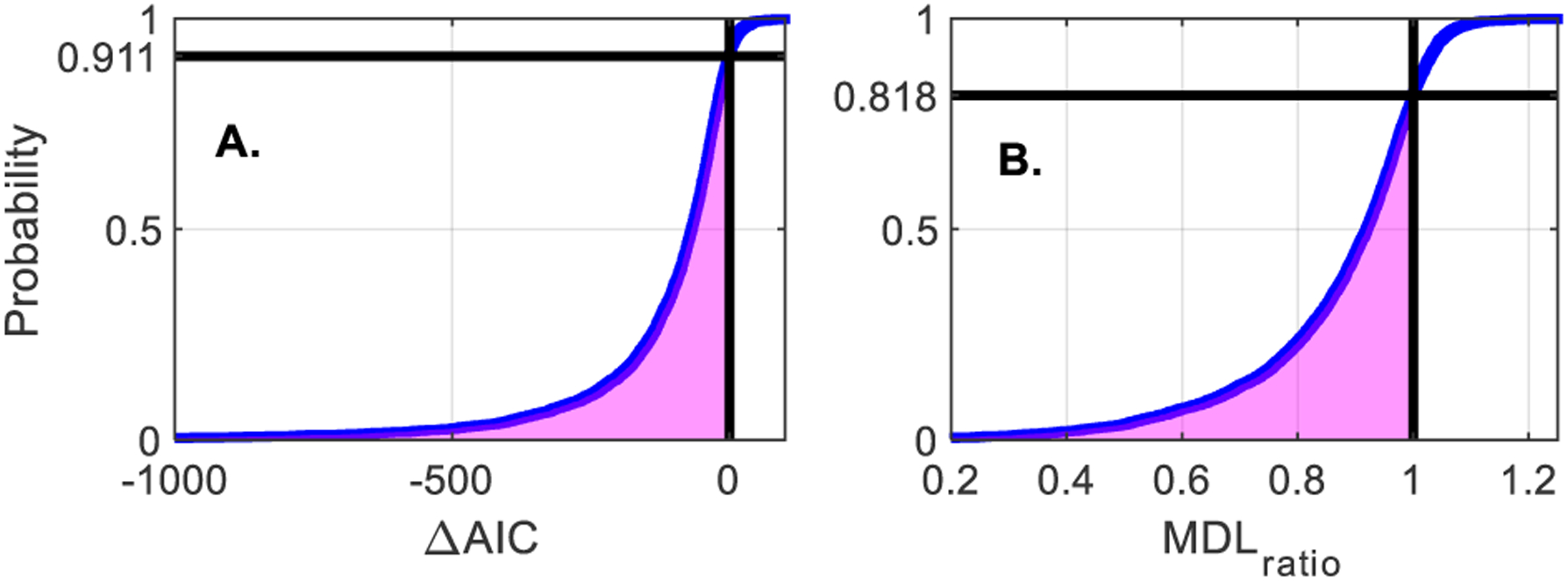
Cumulative distribution of the model selection criteria (A) Δ*AIC* and (B) *MDL*_*ratio*_. The proportion of models for which the NLS was best is in magenta.

Figure 5 shows the cumulative distributions of the Δ*AIC*, and the *MDL*_*ratio*_ criteria comparing the LS and NLS. Figure 5(A) shows that *ΔAIC* was positive, favoring the NLS, in 91.1% of segments. Figure 5(B) shows that the *MDL*_*ratio*_ also favored the NLS, as it was greater than 1 in 81.8% of segments. All segments where the NLS was favored by the MDL were also favored by *ΔAIC*. Thus, MDL was the strictest criterion and determined that in 81.8% of segments, the NLS was the best structure over the LS.

## 4. Discussion

Our major finding was that nonlinear models capture UP-FHR dynamics better than linear models in 81.8% of segments for both the normal and HIE groups. The better performance of a nonlinear UP-FHR model is consistent with known physiological mechanisms. The uterine artery supplies oxygen and nutrients to the placenta and fetus. During labor, uterine contractions reduce the blood flow in the uterine artery causing transitory periods of fetal hypoxia. The fetus protects itself with a series of compensatory mechanisms that cause FHR decelerations [6]. It has been shown that the relationship between the amplitude of uterine contractions and blood flow in the uterine artery is nonlinear [2]. This can be expected to translate in a nonlinear relation between UP and FHR.

An important question is why the LS model performed better than the NLS in 18.2% of the segments. We suspect that this was because for these segments the contractions were not large enough to elicit a nonlinear response. It is difficult to validate this hypothesis because the UP signal is uncalibrated across individuals. This is a limitation of our study that we will address in the future.

Finally, it is important to note that this analysis treated each segment independently. Unless there was a sudden change in the nature of the signals (i.e., a sentinel event or signal disconnection), there is no reason to expect the system parameters to vary widely across consecutive segments. Thus, our current approach does not consider the dependency of the system parameters of one segment on those of adjacent segments. In the future, we will use time-varying methods to study how the system parameters change as a function of time. Also, we will explore the difference of these parameters between the normal and HIE groups.

## 5. Conclusion

We demonstrated that the NLS captured better the UP-FHR relationship during the intrapartum than the LS. When modelling, it is important to have the right model. In this case, since the NLS was better, it means that the parameters of the LS are biased. Reducing the bias in the parameters is important for future attempts to classify which fetuses are progressing towards a normal outcome of labor and those that are at risk of developing HIE.
